# MicroRNA-223 Regulates the Development of Cardiovascular Lesions in LCWE-Induced Murine Kawasaki Disease Vasculitis by Repressing the NLRP3 Inflammasome

**DOI:** 10.3389/fped.2021.662953

**Published:** 2021-05-07

**Authors:** Daisuke Maruyama, Begüm Kocatürk, Youngho Lee, Masanori Abe, Malcolm Lane, Debbie Moreira, Shuang Chen, Michael C. Fishbein, Rebecca A. Porritt, Magali Noval Rivas, Moshe Arditi

**Affiliations:** ^1^Division of Pediatric Infectious Diseases and Immunology, Cedars-Sinai Medical Center, Los Angeles, CA, United States; ^2^Department of Biomedical Sciences, Infectious and Immunological Diseases Research Center, Cedars-Sinai Medical Center, Los Angeles, CA, United States; ^3^Department of Pathology, David Geffen School of Medicine, University of California, Los Angeles, Los Angeles, CA, United States

**Keywords:** miR-223, IL-1 beta, Kawasaki disease, vasculitis, NLRP3, LCWE

## Abstract

Kawasaki disease (KD), an acute febrile childhood illness and systemic vasculitis of unknown etiology, is the leading cause of acquired heart disease among children. Experimental data from murine models of KD vasculitis and transcriptomics data generated from whole blood of KD patients indicate the involvement of the NLRP3 inflammasome and interleukin-1 (IL-1) signaling in KD pathogenesis. MicroRNA-223 (miR-223) is a negative regulator of NLRP3 activity and IL-1β production, and its expression has been reported to be upregulated during acute human KD; however, the specific role of miR-223 during KD vasculitis remains unknown. Here, using the *Lactobacillus casei* cell wall extract (LCWE) murine model of KD vasculitis, we demonstrate increased miR-223 expression in LCWE-induced cardiovascular lesions. Compared with control WT mice, LCWE-injected miR-223-deficient mice (*miR223*^−/*y*^) developed more severe coronary arteritis and aortitis, as well as more pronounced abdominal aorta aneurysms and dilations. The enhanced cardiovascular lesions and KD vasculitis observed in LCWE-injected *miR223*^−/*y*^ mice correlated with increased NLRP3 inflammasome activity and elevated IL-1β production, indicating that miR-223 limits cardiovascular lesion development by downmodulating NLRP3 inflammasome activity. Collectively, our data reveal a previously unappreciated role of miR-223 in regulating innate immune responses and in limiting KD vasculitis and its cardiovascular lesions by constraining the NLRP3 inflammasome and the IL-1β pathway. These data also suggest that miR-223 expression may be used as a marker for KD vasculitis pathogenesis and provide a novel therapeutic target.

## Introduction

Kawasaki disease (KD) was first reported by Tomisaku Kawasaki in Japan, in 1967. KD is a systemic vasculitis and myocarditis of unknown etiology and is the leading cause of acquired heart disease in children in developed countries (1). KD has been reported worldwide; however, its incidence is 10 to 30 times higher among children living in Asian countries or from Asian ancestry compared to those in Europe or in the United States (1–3). Untreated KD can lead to the development of coronary artery aneurysms (CAA) in up to 30% of patients (4). KD treatment consists of a single dose of IVIG (2 g/kg) and aspirin, which reduces the risk of CAA development to ≈4% (1). However, up to 15–20% of IVIG-treated KD patients do not respond to the treatment; these patients develop persistent fevers within 36 to 72 h after the end of IVIG infusion and are at increased risk for developing CAA (5–7). Several alternative treatments such as IL-1 receptor antagonist (anakinra) (8–10), anti-TNF-α antibody (11), corticosteroids (12, 13), and calcineurin inhibitors like cyclosporine (14) have been successfully used to treat IVIG-resistant patients (15).

The NLRP3 inflammasome and IL-1β production have a crucial role in KD pathogenesis. Peripheral blood mononuclear cells (PBMCs) isolated from KD patients spontaneously secrete IL-1β (16), and serum levels of IL-1β are higher during acute KD and decrease markedly during the convalescent phase (17, 18). The expression of IL-1-related genes is also upregulated in PBMCs isolated from KD patients during the acute phase of illness (19, 20), and increased transcript abundance of several genes from the IL-1 pathway is associated with IVIG-resistance (21). Genome-wide association studies led to the identification of single-nucleotide polymorphisms (SNPs) associated with increased susceptibility to KD linked to NLRP3 inflammasome activation and IL-1β production (18, 22, 23). Studies in experimental murine models of KD vasculitis have confirmed those observations and further demonstrated the deleterious role of NLRP3 overactivation and enhanced IL-1β production during KD. Blocking the IL-1 pathway genetically using either *Il1β*^−/−^, *Il1r1*^−/−^, or *Nlrp3*^−/−^ mice, or treating WT mice with either IL-1α- or IL-1β-neutralizing antibodies or IL-1 receptor antagonist, anakinra, significantly reduces vasculitis in the *Lactobacillus casei* cell wall extract (LCWE)-induced KD model (24–26). Similar results were reported with the Candida albicans water-soluble fraction (CAWS) murine model of KD vasculitis (27, 28).

MicroRNAs (miRNAs) are small (20 to 23 nucleotides), endogenous, non-coding RNA molecules that are responsible for various cellular and metabolic pathways, including cell proliferation, differentiation, and death (29). In addition, miRNAs are also involved in the regulation of inflammatory responses and the maintenance of immune homeostasis (30, 31). Indeed, miRNAs control the expression of targeted proteins by either inhibiting mRNA translation or decreasing the levels of their corresponding mRNA (32, 33). Among immune regulatory miRNAs, miR-223, initially identified as specific to the hematopoietic lineage (34), inhibits NLRP3 inflammasome activation in acute lung injury (35) and regulates intestinal inflammation (36). In human KD studies, miR-223 expression is upregulated in KD patients' plasma (37) and whole blood (38), as well as in the coronary artery tissue from autopsy samples collected from children with KD (39).

Although local and systemic miR-223 expression is upregulated during human KD, whether miR-223 prevents or promotes the development of KD coronary arteritis still remains unknown. The objective of this study was to characterize the contribution of miR-223 to the development of LCWE-induced cardiovascular lesions using the well-accepted LCWE-induced murine model of KD vasculitis. We demonstrate that miR-223 expression is upregulated in inflamed abdominal aortic aneurysms and dilatations of LCWE-injected mice. Mice genetically deficient in miR-223 exhibit markedly exacerbated heart vessel inflammation and abdominal aorta aneurysm development, as well as increased levels of circulating IL-1β. Overall, our results support the concept that upregulation of miR-223 in inflamed tissues is beneficial and acts as a feedback mechanism to control the pathological and deleterious overactivation of the NLRP3 inflammasome and subsequent IL-1β production.

## Materials and Methods

### Mice

Wild-type (WT) C57BL/6 and *miR223*^−/*y*^ (B6.Cg*-Ptprc*^*a*^ *Mir223* ^*tm1Fcam*^*/J*) mice were purchased from Jackson Laboratories. For this study, we only used male animals, as LCWE injection induces more severe and more consistent coronary vasculitis lesions and abdominal aorta aneurysms in male than female mice (25, 40). All animals were housed under specific pathogen-free conditions at the animal center of Cedars-Sinai Medical Center. Experiments were conducted under approved Institutional Animal Care and Use Committee protocols.

### Preparation of LCWE

LCWE was prepared as previously described (24). Briefly, *Lactobacillus casei* (ATCC 11578) was grown in Lactobacillus de Man, Rogosa, and Sharpe broth (EMD Millipore, MA, USA) for 48 h, harvested, and washed with PBS. The harvested bacteria were disrupted by an overnight treatment with two packed volumes of 4% SDS/PBS. Cell wall fragments were washed with PBS, and SDS-treated cell wall fragments were sonicated for 2 h with a 3/4-inch horn and a garnet tip at maximum power. During sonication, the cell wall fragments were kept in a dry ice/ethanol bath. After sonication, cell wall fragments were spun for 20 min at 12,000 rpm and 4°C. The supernatant was centrifuged for 1 h at 38,000 rpm and 4°C, and the pellet was discarded. The total rhamnose content of the cell wall extract was determined by a colorimetric phenol-sulfuric assay as described previously (41).

### LCWE-Induced KD Mouse Model

Five-week old male mice were injected intraperitoneally with 500 μg of LCWE (total rhamnose amount as determined above) or PBS. Two weeks later, mice were euthanized and hearts removed and embedded in optimal cutting temperature compound (OCT) for histological examination. For abdominal aorta, diameters were measured at five different parts (below the left renal artery) and maximal abdominal diameters were calculated. In some experiments, aortas were kept in RNA later for RNA extraction. Serial sections (7 μm) of heart tissues were H&E stained and used for pathological examination. Only sections that showed the second coronary artery branch separating from the aorta were analyzed. Histopathological examination and heart vessel inflammation score (coronary arteritis, aortic root vasculitis, and myocarditis) were performed by a pathologist (M.C.F.) blinded to the genotypes or experimental groups, as described previously (24). All images were acquired either with a Biorevo BZ-9000 or BZ-X710 (Keyence) and were further analyzed with ImageJ software.

### RNA Isolation and Quantitative Real-Time PCR

Freshly dissected aortas were stored in RNA later (Qiagen) before RNA extraction. RNA extraction was performed using the miRNEasy micro kit (Qiagen) according to the manufacturer's instructions. Quantitative real-time polymerase chain reaction (qPCR) for miR-223 was performed using the Power SYBR Green RNA-to-Ct 1 step kit according to the manufacturer's instructions (Thermo Fisher Scientific) with the following primer sequences: 5'-TGTCAGTTTGTCAAATACCCCA-3' and 5'-GCGAGCACAGAATTAATACGAC-3', as previously published (42).

### ELISA

IL-1β in serum samples was measured using the U-PLEX Mouse IL-1β Assay (Meso Scale Diagnostics) per the manufacturer's instructions. The samples were read and analyzed by MSD QuickPlex SQ120 instrumentation and Workbench 4.0 Software (Meso Scale Diagnostics).

### Statistical Analysis

Results are reported as mean ± SEM. All data were analyzed using GraphPad Prism software. Statistical significance was evaluated by Student's *t* test (two-tailed) to compare unpaired samples between experimental groups. In experiments where data was not normally distributed, the Mann–Whitney test was performed. A probability value of <0.05 was considered statistically significant.

## Results

### miR-223 Expression Is Increased During LCWE-Induced KD Vasculitis

**Figure 1:**
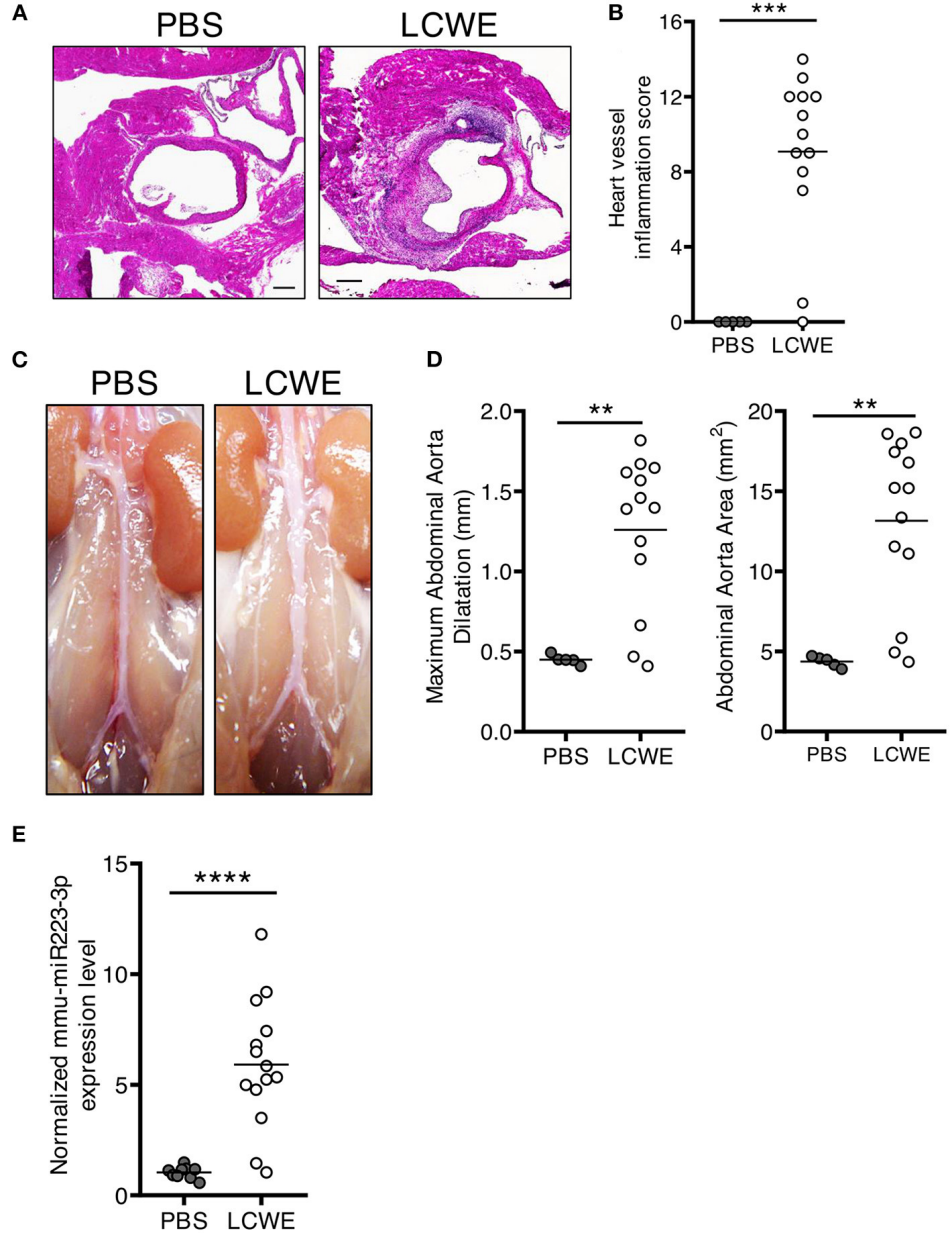
Increased miR-223 expression during LCWE-induced KD vasculitis. Five-week-old mice were injected i.p. with either PBS or LCWE, and 2 weeks later vasculitis severity was assessed. **(A)** H&E staining of heart tissue sections from PBS and LCWE-injected WT mice. **(B)** Heart vessel inflammation score of PBS and LCWE-injected WT mice. **(C)** Pictures of the abdominal aortas of PBS-injected and LCWE-injected mice. **(D)** Maximal abdominal aorta diameter and abdominal aorta area of PBS and LCWE-injected mice 2 weeks post-LCWE injection. **(E)** miR-223 mRNA quantification in the abdominal aorta of PBS and LCWE-injected mice. ^**^p < 0.01, ^***^p < 0.001 and ^****^p <* 0.0001* by unpaired *t*-test.

As compared with PBS-injected WT control mice, LCWE injection resulted in the development of heart inflammation manifested by aortitis and coronary artery aneurysms (Figures 1A,B). LCWE-injected mice also exhibited prevalent abdominal aorta aneurysms and dilatations (Figures 1C,D). Elevated levels of miR-223 have been reported in the serum of acute KD patients (43), and miR-223 expression is increased in coronary artery aneurysms of KD children. To assess miR-223 expression in the mouse model, we isolated RNA from the abdominal aortas of control mice and LCWE-injected KD mice and measured miR-223 expression by quantitative PCR. Compared with PBS-injected control mice, miR-223 expression levels were significantly higher in abdominal aorta aneurysms of LCWE-injected mice (Figure 1E).

### Exacerbated LCWE-Induced Cardiovascular Lesions in miR-223^–/y^ Mice

**Figure 2:**
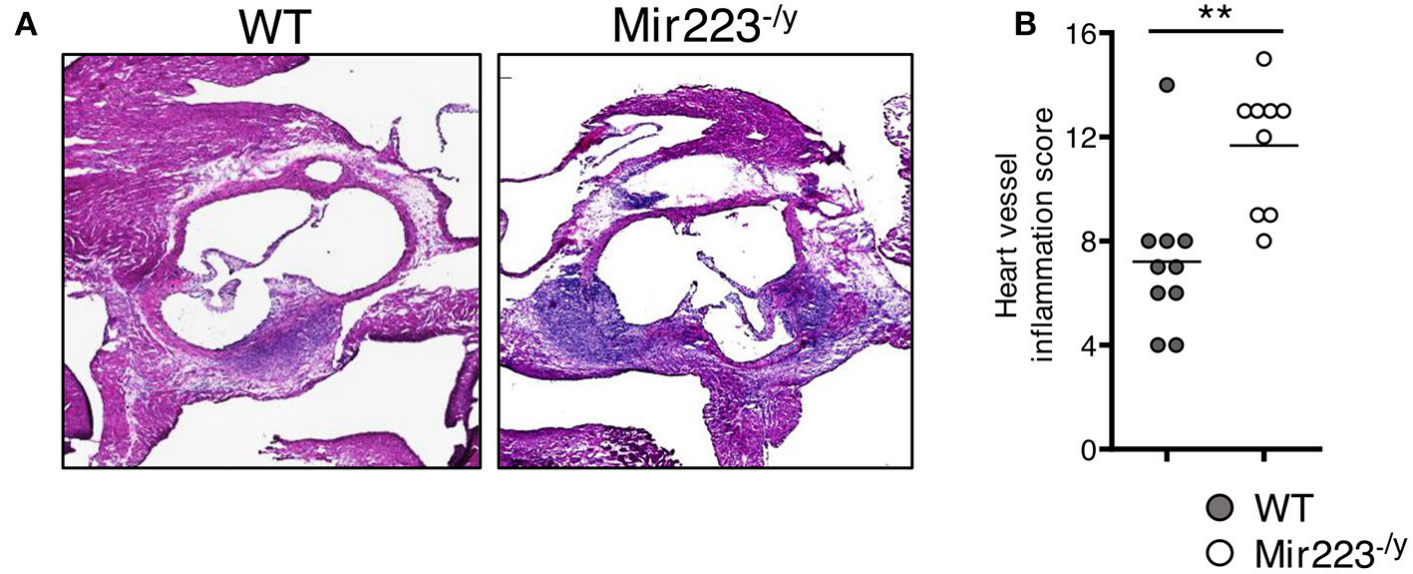
Deletion of miR-223 promotes the development of LCWE-induced vasculitis. Five-week-old male WT and *miR-223*^−/^^y^ mice were i.p. injected with LCWE, and 2 weeks later the severity of vasculitis was assessed. **(A)** H&E heart tissue sections of LCWE-injected WT and *miR-223*^−/^^y^ mice. **(B)** Heart vessel inflammation score of the mice group **(A)**. ^**^p <* 0.01* by unpaired *t*-test.

**Figure 3:**
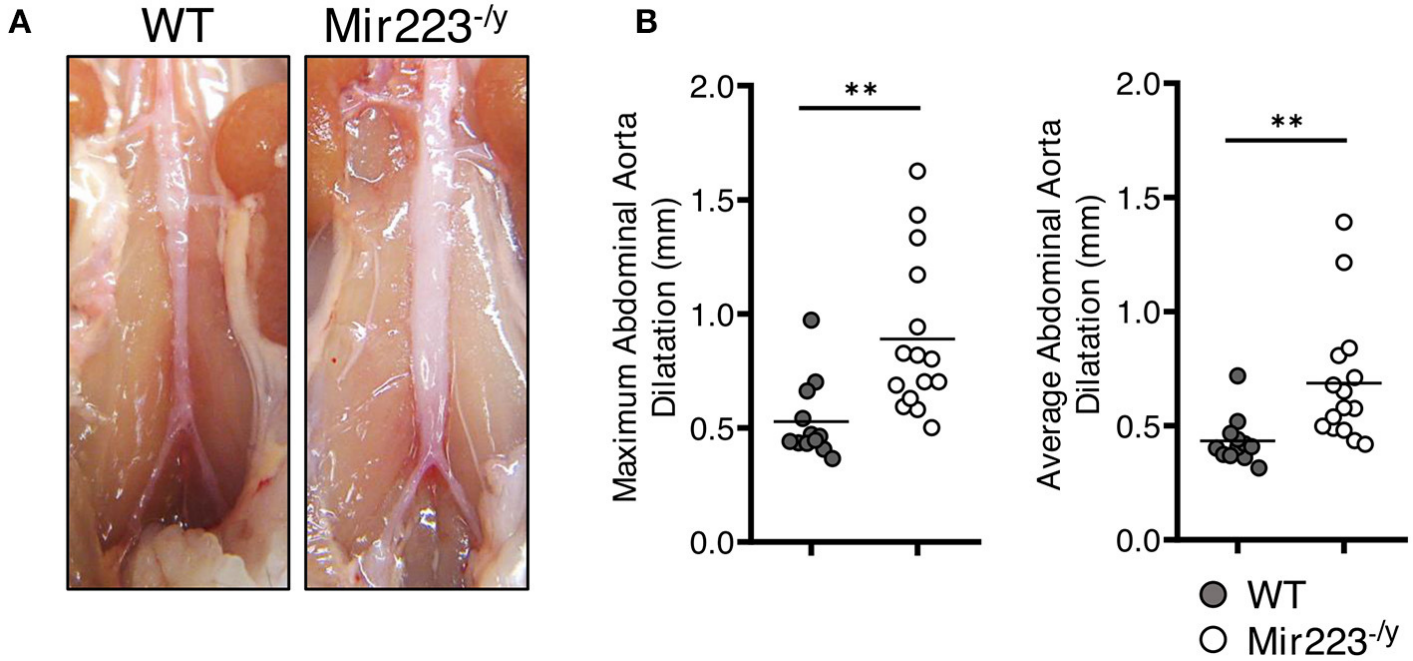
Deletion of miR-223 promotes the development of LCWE-induced abdominal aortitis. Five-week-old male WT and *miR-223*^−/^^y^ mice were i.p. injected with LCWE, and 2 weeks later the severity of vasculitis was assessed. **(A)** Abdominal aorta pictures of LCWE-injected WT and *miR-223*^−/^^y^ mice. **(B)** Maximal abdominal aorta diameter and average abdominal aorta diameter from WT and *miR-223*^−/^^y^ mice injected with LCWE. ^**^p <* 0.01* by unpaired *t*-test.

To determine the effect of miR-223 on the development of cardiovascular inflammation, we injected WT or *miR223*^−/*y*^ mice with either PBS or LCWE and assessed the severity of LCWE-induced KD vasculitis 2 weeks later. Compared with WT mice, LCWE-injected miR-223-deficient mice showed significantly more severe heart vessel inflammation and coronary arteritis (Figures 2A,B). Similarly, deletion of miR-223 also resulted in enhanced development of abdominal aorta aneurysms (Figure 3A) and greater aortic dilations and maximal abdominal aorta diameter (Figure 3B). miR-223-deficient mice injected with PBS did not have any cardiovascular lesions and were similar to the WT mice injected with PBS (data not shown). Overall, our results strongly indicate that miR-223 is required to control the severity and development of LCWE-induced cardiovascular lesions, and miR-223 deletion results in worsened heart and abdominal aorta inflammation.

### miR-223 Regulates LCWE-Induced Vasculitis Induction by Regulating IL-1 Production

**Figure 4:**
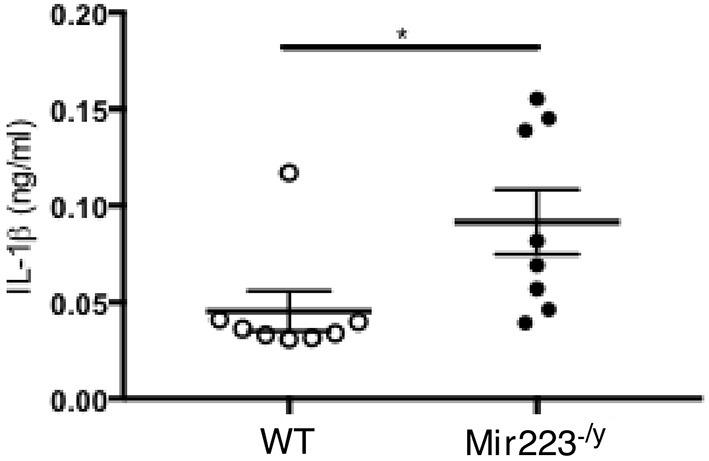
miR-223 dampens LCWE-induced vasculitis induction by regulating IL-1β signaling. IL-1β quantification in the serum of WT and *miR-223*^−/*y*^ at 1 week post-LCWE injection. ^*^p < 0.05 by unpaired *t*-test.

LCWE-induced KD vasculitis is NLRP3 dependent, and it has been previously shown that miR-223 regulates NLRP3 activation and IL-1β production (36). To further determine if miR-233 dampens LCWE-induced KD vasculitis in WT mice by decreasing NLRP3 inflammasome activation and subsequent IL-1β production, we next quantified the circulating levels of IL-1β in LCWE-injected WT and *mir-223*^−/*y*^ mice 1 week after LCWE injection. In agreement with our observation of heightened heart vessel inflammation and increased severity of abdominal aorta aneurysms in LCWE-injected *miR223*^−/*y*^ mice (Figures 2, 3), circulating IL-1β was markedly higher in the absence of miR223 (Figure 4). Overall, our results indicate that miR-223 does not completely prevent the development of LCWE-induced cardiovascular lesions; however, by restraining NLRP3 activation and its subsequent IL-1β production, miR-223 appears to curtail vascular tissue inflammation.

## Discussion

MicroRNAs (miRNAs) are critical regulators of a host of cellular processes, including inflammation (30, 31). These endogenous, non-coding, single-stranded RNAs of 20–23 nucleotides exert regulatory functions through complementary base pairing to the 3′ untranslated regions (UTRs) of protein-coding mRNAs. Many immune processes are regulated by miRNA-mediated RNA interference, but how miRNA circuits orchestrate aberrant cardiovascular inflammation during KD vasculitis is poorly defined. The role of miRNAs in regulating innate immune responses has primarily been investigated for TLR signaling (44). Indeed, several miRNAs were identified as induced following TLR activation, targeting mRNAs encoding components of the TLR-signaling pathway itself. These regulatory systems likely evolved to allow a strong initial immune response that must be gradually dampened down after the secondary induction of the regulatory miRNAs. miRNA-223 is a critical regulator of NLRP3 inflammasome activity (42). miR223 suppresses NLRP3 expression through a conserved binding site within the 3′ untranslated region of NLRP3, resulting in reduced NLRP3 inflammasome activity (42). Thus, miR-223 functions as an additional layer of control beyond the tight transcriptional control of NLRP3 message itself. miR-223 also plays a key role in the regulation of granulocyte differentiation, among other functions (34).

Changes in the expression of multiple miRNAs are reported during KD; however, whether these differentially expressed miRNAs have a beneficial or deleterious role on KD pathogenesis remains unknown. Shimizu et al. reported that six microRNAs (miRs-143,−199b-5p,−618,−223,−145, and−145) were significantly elevated in whole blood of acute KD patients (38). Elevated miR-145 expression in blood samples from acute KD patients was further confirmed by qRT-PCR in an independent cohort. miR-145 may play a critical role in the differentiation of neutrophils and vascular smooth muscle cells by modulating TGF-β signaling in the arterial wall (38). Yun et al. reported that the serum levels of miRNA-200c and miRNA-371-5p were elevated during KD and could potentially be used as a KD diagnostic biomarkers (45). Rong et al. suggested that miR-27b could affect endothelial cell proliferation and migration *via* targeting Smad7 and affecting TGF-β pathway. Thus, miR-27b may be a potential biomarker for KD and a therapeutic target for KD treatment (46). In another study, seven miRNAs were significantly upregulated (hsa-let-7b-5p, hsa-miR-223-3p, hsa-miR-4485, hsa-miR-4644, hsa-miR-4800-5p, hsa-miR-6510-5p, and hsa-miR-765) and three were significantly downregulated (hsa-miR-33b-3p, hsa-miR-4443 and hsa-miR-4515) in acute KD compared with the healthy controls by miRNA microarray analysis (37). Of these miRNAs, miR-223 was consistently detected by RT-qPCR (37), and previous studies have reported that miR-223 may regulate inflammation of vascular endothelial cells (47).

**Figure 5:**
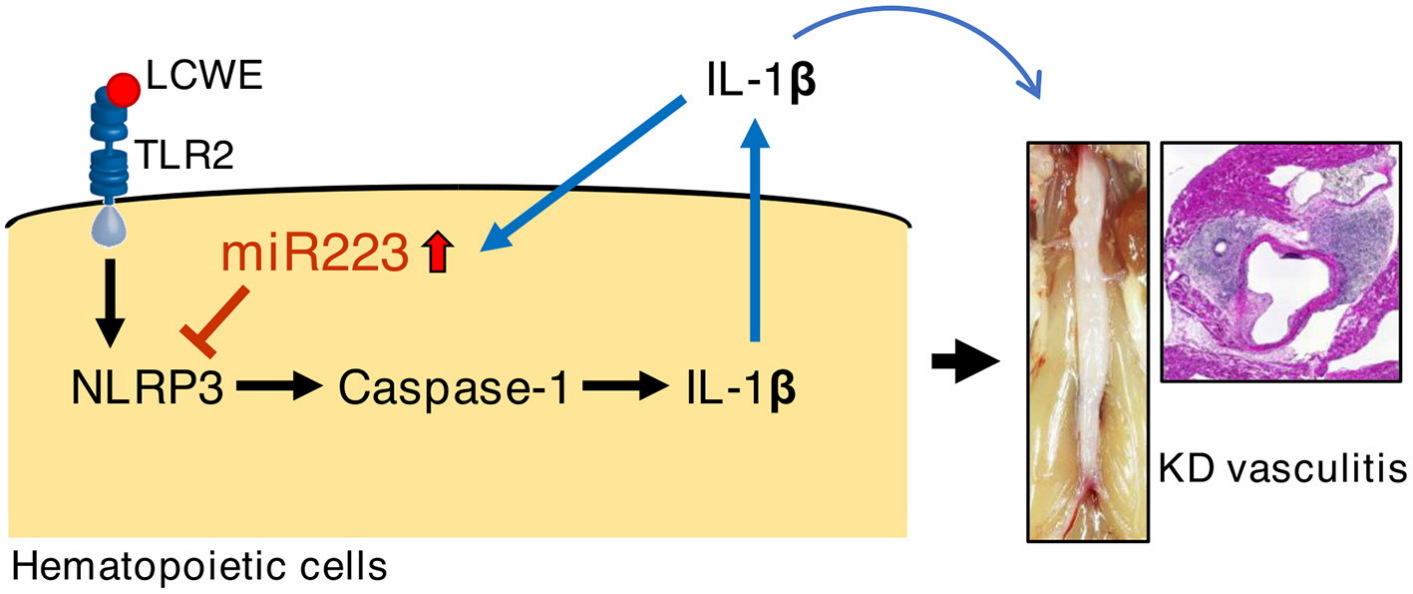
Possible mechanism of miR-223 regulation during LCWE-induced vasculitis.

The role of miR-223 in regulating innate immune response and inflammation is increasingly appreciated. Absence of miR-223 exacerbates inflammation in a murine model of colitis characterized by enhanced NLRP3 inflammasome activation and IL-1β production (36). LPS and poly(I:C) activation decrease miR-223 expression in macrophages through TLR4 and TLR3. In turn, downregulation of miR-223 promotes TNF-α, IL-6, and IL-1β production upon LPS stimulation (42, 48). miR-223 has also been shown to disrupt NLRP3 inflammasome activity in a mouse model of hepatitis (49). In acute gout patients, colchicine can upregulate miR-223-3p and downregulate IL-1β in the plasma (50). LPS can reduce miR-223, while promoting the production of IL-1β in human adipose stem cells *via* TLR2 (51). Previously, we reported that macrophages infiltrate the cardiovascular lesions of LCWE-injected mice (25, 26). These infiltrating macrophages show high caspase-1 activity by FLICA staining (26). In this study, we found that miR-223 was highly expressed in the abdominal aorta of LCWE-injected mice. Moreover, *miR-223*^−/*y*^ mice showed more severe abdominal aorta dilatations and heart vasculitis and higher concentrations of serum IL-1β, which is a key mediator of LCWE-induced KD vasculitis (24, 25). We hypothesize that intense IL-1β production during LCWE-induced KD vasculitis induces miR-223 expression as a negative regulatory feedback loop to suppress IL-1β function and further development of vasculitis (Figure 5).

Johnnidis et al. reported that miR-223 negatively regulates granulocyte differentiation and dampens neutrophil activation and effector functions (34). Indeed, *miR-223*-deficient mice spontaneously develop lung pathologies due to increased numbers of granulocyte progenitors and neutrophils, which are more easily activated (34). Additionally, Dorhoi et al. reported that in an experimental murine model of *Mycobacterium tuberculosis* infection, miR-223 deletion resulted in exacerbated neutrophil-driven lung inflammation (52). We have reported that Ly6G^+^ neutrophils infiltrate the abdominal aorta aneurysm area of LCWE-injected WT mice (25), and similar results were also observed with the CAWS murine model of KD vasculitis, where a massive influx of Ly6G^+^ neutrophils is detected in the coronary artery of WT mice 28 days after CAWS injection (53). Since miR-223 regulates neutrophil differentiation and activity, it is possible that hypersensitive and activated neutrophils participate and enhance the severity of cardiovascular lesions observed in LCWE-injected *miR-223*^−/*y*^ mice; however, future studies will be needed to investigate the role of miR-223 in modulating neutrophil activity in this experimental model. In addition, human platelets also express significant amounts of miR-223, and platelet miR-223 levels are important for their reactivity (54). The lower expression of miR-223 may increase platelet reactivity and the risk of thrombotic disease, such as myocardial infarction (55). In the acute stage of KD, patients show thrombosis and decreased platelet numbers, and then thrombocytosis is consistently found in the 2nd to 3rd week of illness (56). Lack of platelet-derived miR-223 in KD patients may increase the risk of coronary artery pathology (43). Recent studies have also shown that leukocytes and platelet-secreted miR-223 can enter vascular smooth muscle cells, and appear to play important protective roles in their function in experimental models of atherosclerosis (57), in an arterial injury repair model (58), and in a KD mouse model by decreasing VSMC proliferation (59).

Although miR-223 has been reported to negatively control NLRP3 activation (42), it is also possible that miR-223 targets other proteins involved in inflammatory immune responses that may potentially contribute to LCWE-induced KD vasculitis, such as CXCL2, CCL3 (52), STAT3, and IL-6 (60). While we hypothesize that in this model miR-223 affects the NLRP3 and IL-1β pathway, the specific mechanisms of this regulation will need to be further demonstrated in future experiments.

## Conclusions

Collectively, our studies highlight the miR-223-NLRP3-IL-1β regulatory circuit as an important component of vascular inflammation development in the experimental LCWE-induced murine model of KD vasculitis. Our data reveal a previously unappreciated role of miR-223 in regulating the level of NLRP3 inflammasome activation and shows that miR-223 provides an early break, limiting IL-1β-mediated vascular inflammation in LCWE-injected mice. NLRP3 is under a tight transcriptional control, and miR-223 has been shown to work as an important rheostat controlling NLRP3 inflammasome activation (42). Our observation that miR-223 deficiency results in a more severe LCWE-induced KD vasculitis is therefore consistent with this previous report and indicates that a similar control of inflammation may indeed occur in the LCWE-induced KD murine model. Hence, miR-223 may be a potential biomarker for early diagnosis of human KD, and as miR-223 can dampen cardiovascular inflammation, pharmacologic stabilization of miR-223 may hold promise as a future novel therapeutic modality for KD.

## Data Availability Statement

The raw data supporting the conclusions of this article are available from the corresponding author upon reasonable request.

## Ethics Statement

All experiments were performed according to Cedars-Sinai Medical Center Institutional Animal Care and Use Committee (IACUC) guidelines.

## Author Contributions

MAr conceived and supervised the project. MAr, YL, and MNR wrote the manuscript. DMa, BK, YL, MAb, ML, DMo, SC, MCF, and RAP performed all of the experiments. SC, RAP, MNR, and MAr provided critical editing and content to the manuscript as well as experimental design. All authors read and approved the final manuscript.

## Conflict of Interest

The authors declare that the research was conducted in the absence of any commercial or financial relationships that could be construed as a potential conflict of interest.
